# Refugees’ integration and emotional distress over the course of 9 months

**DOI:** 10.3389/fpsyg.2024.1459934

**Published:** 2024-10-17

**Authors:** Flurina Potter, Katalin Dohrmann, Brigitte Rockstroh, Anselm Crombach

**Affiliations:** ^1^Department of Psychology, University of Konstanz, Konstanz, Germany; ^2^Vivo International, Konstanz, Germany; ^3^Department of Psychology, Saarland University, Saarbrücken, Germany

**Keywords:** refugees, mental health, integration, emotional distress, longitudinal research, follow-up study, Germany

## Abstract

**Background:**

High prevalence rates of mental disorders are reported in refugees due to experiencing substantial pre-, peri-, and post-migration stress. While long-term studies indicated that emotional distress of refugees either stagnates or ameliorates over time, long-term research on refugees’ integration and its’ interaction with emotional distress is limited. The examined long-term predictors for refugees’ emotional distress and integration in this study were, amongst others, severe physical abuse in childhood, residence status and length of stay.

**Methods:**

The sample included 46 refugees, 91.3% male, mean age 20.8 years. Trained psychologists screened for emotional distress with the use of the Refugee Health Screener in a semi-structured interview. Integration progress was screened using the Integration Index with the subdimensions psychological, economic, political, social, linguistic and navigational integration. Longitudinal differences for emotional distress and integration sub-dimensions were evaluated by sign tests and t-tests. The longitudinal course of integration was evaluated with a Mixed ANOVA. Further, two hierarchical regression analyses were performed to analyze longitudinal predictors of emotional distress and integration.

**Results:**

Overall, emotional distress decreased, and integration increased over time. In particular, the sub-dimensions of social, economic, and linguistic integration increased significantly over time_._ Two regression analyses determined significant predictors of (a) emotional distress_t1_ (adjusted *R^2^* = 0.46): psychotherapy (*ß* = 0.35, *p* = 0.020), emotional distress_t0_ (*ß* = 0.34, *p* = 0.031), and integration_t0_ (*ß* = −0.29, *p* = 0.043), and one significant predictor of (b) integration_t1_ (adjusted *R^2^* = 0.70): integration_t0_ (*ß* = 0.89, *p* < 0.001).

**Conclusion:**

This is the first study to longitudinally examine the Integration Index with all subdimensions. Over the course of 9 months refugees’ overall integration, and the sub-dimensions of social, economic, and linguistic integration increased. Whilst the emotional distress of initially highly distressed refugees ameliorated over the course of nine months, their symptom severity remained clinically significant. Results emphasize the importance of early integration for the long-term development of mental health and integration in refugees. Refugees’ emotional distress and integration are intertwined and need to be addressed promptly after refugees’ entry into the host country.

## 1 Introduction

In recent years, Germany has become one of the major host countries for refugees. In 2021, Germany hosted around 2.6 million refugees (United Nations High Commissioner for Refugees, 2021) and in 2023, Germany received the second highest number of new individual asylum applications, the world over (United Nations High Commissioner for Refugees, 2024). Refugees experience substantial pre-, peri-, and post-migration stress (Bogic et al., 2015). Numerous studies identified these stressful experiences as sources of emotional distress and cause of a high prevalence of mental disorders (Schick et al., 2016; Kartal et al., 2018). In addition, integration has been shown to be challenging for states, societies and health care systems (Schick et al., 2016; Kaltenbach, 2019; Silove et al., 2017) and existing approaches have often not been sufficient (German National Academy of Sciences Leopoldina, 2018; Walther et al., 2021). Further evidence of intensity and course of emotional distress in refugees, as well as the impact of emotional distress on refugees’ integration, could provide useful information for integration programs. Refugees’ access to health care service in Germany is limited and only few get the recommended treatment (German National Academy of Sciences Leopoldina, 2018; Dumke et al., 2024; Schneider et al., 2017). Structural barriers include that despite European guidelines and research recommendations a general mental health screening for all arriving refugees has not yet been implemented (German National Academy of Sciences Leopoldina, 2018; Commission E, 2018) and that during the first 18-months^1^, most asylum seekers in Germany did not have regular health insurance. Result is that only treatment for pain and acute illnesses, vaccinations, emergency and maternity care is covered (Bauhoff and Göpffarth, 2018; Baron and Flory, 2020). Additional services -such as psychotherapy- must be approved by municipalities on a case-by-case basis (Bauhoff and Göpffarth, 2018). The present study follows a cross-sectional assessment, conducted from 2020 to 2022, in Germany (Potter et al., 2022) that assessed adolescent refugees’ and asylum seekers’ emotional distress and integration. Subsequently, refugees^2^ in need of psychotherapy were referred to psychotherapist within the German health care system. Nine months after the initial assessment the intensity of emotional distress and integration was once again evaluated to assess changes and their reciprocal impact on each other. This research was conducted in a project facilitating adolescent refugees’ access to mental health services in Germany.

A recent meta-analysis emphasized the high prevalence of stress- and trauma-induced mental disorders in refugee populations (Hoell et al., 2021). Prevalence rates for newly arrived refugees in Germany were reported as high as 29.9% for post-traumatic stress disorder (PTSD), and 39.8% for depression. In adolescent refugees in Europe, Kien et al. (2019) found that up to 50% met the criteria for PTSD. Moreover, up to one-third of the sample presented with emotional or behavioral problems or met criteria for anxiety disorders or depression. Longitudinal studies examining *emotional distress* in refugees over varying time intervals documented stagnating as well as ameliorating emotional distress. For instance, Borho et al. (2020) reported relatively stable emotional distress levels over a course of one and a half years in a German sample. Similarly, Jakobsen et al. (2017) found refugees’ emotional distress levels to stay relatively unchanged over 26 months in a Norwegian sample. However, whilst Nikendei et al. (2019) reported that whilst posttraumatic stress and general anxiety in refugees in Germany did not significantly change over a three-month course, they observed that panic symptoms, depression, and quality of life scores improved. In accordance, a decline of psychiatric diagnoses - except PTSD - was found over a course of four to ten months in refugees in Germany (Richter et al., 2018). In refugees in Australia, psychological distress and PTSD symptoms improved over a 5 years period (Stuart and Nowosad, 2020).

Differences in the course of emotional distress may vary with social circumstances and living conditions (Richter et al., 2018) and research has proposed a dose–response relationship of post-migration stress (Ryan et al., 2008). Cross-sectional analyses elucidated the interplay of emotional distress and *integration:* Successful integration was found to be the best acculturation strategy for emotional well-being, followed by assimilation, marginalization, and separation (Behrens et al., 2015; Han et al., 2016). Research has repeatedly shown that refugees with mental disorders struggled to engage in integration activities, such as seeking employment or forming relationships with the host population (Phillimore, 2011). In interviews in Germany, refugees reported an interaction between their emotional distress and integration efforts across different areas of integration, e.g., how they felt overwhelmed by bureaucracy (Walther et al., 2021). Moreover, learning a new language was described to have taken a mental toll for some, e.g., for illiterate refugees, or when worries about the family that was left behind were too pressing (Walther et al., 2021). Further, they reported how mental health problems rooted in past traumata, with symptoms such as intrusions, sleeping or concentration problems, may hinder their abilities to visit an integration course or integrate into the German society (Walther et al., 2021). In this study, we follow the definition of (Harder et al., 2018), defining integration “as the degree to which immigrants have knowledge and capacity to build a successful, fulfilling life in the host society.” In this context, knowledge was defined as the skill to navigate the labor market of the host country whilst comprehending the host country’s national language, social institutions, and political system; further capacity was defined as the immigrants’ mental, social and economic assets, which might be useful to invest in their future development in the host country (Harder et al., 2018). In line with this definition integration was subdivided into six different dimensions: psychological, economic, political, navigational, social, as well as linguistic integration (Harder et al., 2018). Filling the lack of a common empirical measure of immigrant integration, the integration index allows comparisons across different immigrant groups (Harder et al., 2018). Evaluating the empirical performance of the integration index, Harder (Harder et al., 2018) completed four surveys with four different immigrant groups. They showed that the measure distinguished among these groups. In the United States a stratified sample of high-income immigrants showed an average standardized integration score of 0.8, a sample of low-income immigrants a score of 0.55, and in immigrants recently enrolled in English language classes the score was 0.46. Moreover, in a stratified sample of immigrants in Germany the score was 0.69.

Longitudinal studies examining an interplay between emotional distress and integration confirmed post-migration stressors such as loneliness, social integration stressors (Chen et al., 2019) and economic stressors (Wu et al., 2021) as relevant risk factors for refugees’ mental health. Similarly, Stuart and Nowosad (2020) found post-migration stressors such as cultural integration, financial stress and loneliness to affect refugees’ mental health more than pre-migration stressors over a course of 5 years. Moreover, Beiser (2006) showed that linguistic integration had more influence on refugees’ mental health in their later phase of resettlement than in their early phase of resettlement. However, in turn, increased psychological distress 3 years post-migration was shown to predict acculturative difficulties 20 years later (Tingvold et al., 2015). Lichtenstein and Puma (2019) examined different integration pathways in a sample of newly arrived refugees over a 4-year period in the USA and found that integration steadily increased. They further concluded that integration dimensions may evolve differently depending on the resettlement stage: While the pathways *employment and economic sufficiency* and *language and cultural knowledge* only increased in the first year of resettlement, the *social bonding* and *social bridging* pathways increased in the first and third year. Contrary to that, Schick et al. (2016) examined refugees seeking psychological treatment in outpatient clinics in Switzerland and concluded that they showed serious integration difficulties regardless of their duration of stay. Overall, longitudinal research on the detailed course of integration is still limited, and regarding subdimensions of integration almost non-existent.

Pre-migration stressors, particularly a high trauma load, were frequently associated with an elevated risk for stress related mental disorders (Kartal et al., 2018; Steel et al., 2009; Wilker et al., 2015). Especially interpersonal violence (Tinghög et al., 2017), physical abuse and other types of abuse during childhood (Margolin and Vickerman, 2011; Lindert et al., 2014) showed strong associations with mental health problems. A national cohort study accomplished by Webb et al. (2017) indicated that trauma-related hospital admissions, related to interpersonal violence or self-harm, increased the risks for later self-harm and violent criminal offending at ages 15–35 years. Hence, another factor adding to the dose–response relationship of trauma and emotional distress over time could be *severe physical abuse in childhood*, a pre-migration stressor with longitudinal prognostic value (Webb et al., 2017).

Regarding the impact of the *residence status*, studies in Australia (Silove et al., 2007), Ireland (Ryan et al., 2008), and Norway (Jakobsen et al., 2017) showed that the refusal of asylum was associated with higher levels of emotional distress over 11–26 months. Nevertheless, two reviews came to the conclusion that residence status was not a reliable predictive factor for unaccompanied refugee minors’ mental health (Höhne et al., 2020; Hornfeck et al., 2022). As another post-migration stressor, the *COVID-19 pandemic* has been shown to affect psychiatric disorders in refugees (Alpay et al., 2021). Moreover, several studies showed that the COVID-19 pandemic exacerbated PTSD symptoms in refugees (Kizilhan and Noll-Hussong, 2020). On top of other pre- and post-migration stressors, negative consequences of the pandemic added more strongly to the emotional distress of refugees compared to the general population (Kizilhan and Noll-Hussong, 2020; Aragona et al., 2020; Gibson et al., 2021).

Taken together, accumulating evidence indicated that emotional distress and integration within the first post-migration years are relevant predictors for the long-term integration and adaption of refugees to their new lives. A more thoroughly understanding of their interplay could help to prevent a vicious cycle between poor mental health caused from pre- and peri-migration traumata aggravated by post-migration stressors (Walther et al., 2021). Yet, integration has so far mainly been assessed in relation to social, cultural, economic, and linguistic integration. Evidence on emotional distress and its interplay with the integration status is limited, particularly of adolescent refugees. During the first cross-sectional assessment, we identified the time living under the restrictions of the pandemic and severe physical abuse in childhood as crucial predictors for emotional distress (Potter et al., 2022). Moreover, the length of stay in Germany as well as emotional distress were identified as most important predictors of integration. Aiming to assess the longitudinal impact of these factors on mental health and successful integration in refugees, the present study focused on the interplay of emotional distress and integration over the course of 9 months.

## 2 Methods

### 2.1 Sample

Participants were invited to partake in the initial assessment (assessment_t0_) if they met the inclusion criteria of refugee status and presented sufficient understanding of study procedures and questions. The assessment_t0_ of 104 refugees took place from March 2020 to June 2022. Follow-up assessments (assessment_t1_) were scheduled 9 months after the assessment_t0_. We tried to contact all participants of the assessment_t0_ and 53 refugees took part in the assessment_t1_ from April 2021 to March 2023. The most common reason for dropouts was that participants could not be reached because they had moved, had been deported or their contact details were missing. Other refugees were contacted but refused to participate for reasons of time or motivation. Thus, the final dropout rate was 44.9%. In addition, data of one refugee aged 41 years was excluded due to the research’s focus on refugee adolescents. From the sample of assessment_t1_ completers, data of *n* = 6 participants had to be excluded due to missing data on one of the key variables emotional distress or integration. This resulted in an assessment_t1_ total sample of *N* = 46, which included 42 male (91.3%) and 4 female (8.7%) participants. Age at assessment_t1_ ranged from 15 to 23 years, with a mean age of 20.8 (*SD* = 2.2) years. In case of missing socio-demographic data, missing values are indicated by the deviating *n* in the results section. Assessment_t1_ participants and dropouts were comparable in most aspects of the assessment_t0_ (see Supplementary material 1), yet dropouts reported possessing health insurance cards more often.

### 2.2 Design and procedure

The research was conducted in a model project called “Fearless,” implemented by the Center of Excellence for Psychotraumatology of the University of Konstanz, the Bodensee-Institut für Psychotherapie, and the NGO “vivo international.” The overall aim of the project is facilitating adolescent refugees’ access to mental health services in Germany. Translators and peer counsellors were trained in translation in psychosocial contexts, mediating between cultures and navigating the German health care system. Therapists received 4 days of training in the diagnosis and treatment of post-traumatic disorders using Narrative Exposure Therapy (NET) and Forensic Offender Rehabilitation NET (FORNET), an adaptation of NET for offenders with a low aggression threshold (Hecker et al., 2015). Thereafter, therapists were supervised by psychotherapist supervisors in the German health care system who received project supervision in NET and FORNET if needed. Psychologically distressed refugees were offered psychotherapy – not limited to trauma-focused treatments - within the project or were referred to other services. For more information on the treatment see Potter et al. (2023). For the assessment_t0_, government authorities working with refugees in the German state of Baden-Württemberg, mostly near the city of Konstanz, were informed of the upcoming research project. Social workers employed at the refugees’ accommodation informed potential participants about the project. Targeted participants were between 14 and 22 years of age. Appointments for the assessment_t1_ were mostly scheduled via the social worker, in some cases the assessment_t1_ was scheduled via the therapist or directly with the participants themselves.

Most of the assessments took place in the refugees’ accommodation in a separate office, some took place in the office of the Center of Excellence for Psychotraumatology at the University of Konstanz. During both assessments, questionnaires were administered in a semi-structured interview. In the assessment_t0_ trained interpreters were present in 50% and in the assessment_t1_ in 35% of all cases. The remaining assessments were conducted in English or German. Prior to both assessments, participants were informed about the study and signed a written informed consent form. For minors, a legal guardian gave additional written consent. In both assessments we explained that the participation was voluntary, that it would not influence the asylum procedure and that all data would be handled confidentially. In the assessment_t0_ no monetary compensation was offered while for assessment_t1_ participants received 15€ for participating. The assessment_t0_ lasted about 45 min and the assessment_t1_ about 40 min. After completion of the assessment_t0_, the refugees’ symptoms and potential treatment offers were discussed, which included psychotherapy within the project or transfer to other institutions. After assessment_t1_ all emotionally distressed participants who had not yet received therapy were offered assistance to get access to treatment. They were informed that they would not have to pay for any treatment and that it was voluntary. The project and current study were approved by the Ethics Committee of the University of Konstanz.

### 2.3 Assessment

The assessment_t0_ included *sociodemographic information* about age, country of origin, years of education, residence status, health insurance, arrival date in Germany, whether the participants had arrived accompanied by family members and whether they had family living in Germany. Moreover, they were asked if they had been involved in physical fighting since entering the country. In addition to sociodemographic information, *length of stay* was calculated according to the arrival date in Germany and *pandemic months* was calculated according to the months passed since March 2020. *Severe physical abuse in childhood* was assessed with the question: “Have you been hit so badly before the age of 15 that you had to go to hospital or needed medical attention?” Participants responded with a yes/no answer. This question refers to the findings of Webb (Webb et al., 2017), who analyzed hospital admissions in a national cohort study, and showed that having experienced interpersonal violence causing hospitalization prior to the age of 15 years considerably increased the risk of harming one-self and/or being convicted because of a violent crime between the age of 15–35 years.

In the assessment_t1_, participants were asked about their present residence status, health insurance, vocational training and German language skills. Moreover, they were asked if they had been involved in physical fighting or had started psychotherapeutic treatment since the assessment_t0_. Participants who had started therapy were asked when it had started, how many outpatient sessions had been held, and if they had further remarks regarding therapy. The following questionnaires were administered in both assessments:

The *Refugee Health Screener* (RHS) was used to assess refugees’ *emotional distress* (Hollifield et al., 2013). The questionnaire shows good reliability and concurrent and predictive validity for anxiety, depression, and PTSD and has been used in diverse settings with refugees (Hollifield et al., 2013; Kaltenbach et al., 2017; Hollifield et al., 2016). The present study used the RHS-13 with 13 items (Hollifield et al., 2016), which proved to be efficient without compromising specificity or sensitivity while having acceptable psychometric properties (Hollifield et al., 2016). Participants rated the degree to which they were troubled by a symptom in the last month, e.g., “feeling down, sad, or blue most of the time.” The items were rated from 0 (*not at all*) to 4 (*extremely*) on a 5-point Likert scale and the cumulative score ranges between 0 and 52. Participants (*n* = 3) that did not rate > 10% of the RHS-13 items were excluded from the analyses (Hollifield et al., 2016). If participants’ missing items accounted for ≤10% of the RHS-13 (Kaltenbach et al., 2017), the respective items were set to zero. We used a dichotomous cut-off score. Psychotherapy was offered to participants with a total score ≥ 11, indicating high emotional distress (Hollifield et al., 2016). In the present study, Cronbach’s alpha for the instrument resulted in α_t0_ = 0.91 (*n* = 44) and α_t1_ = 0.87 (*n* = 44).

The *Integration Index,* developed by the Immigration Policy Lab (IPL), was used as a measure of the refugee’s status in his/her integration process. The IPL questionnaire assesses psychological, economic, political, social, linguistic and navigational dimensions of *integration* (Harder et al., 2018). The IPL has proven valid across immigrant groups and countries and correlates with length of stay and residence status and differentiates among groups with diverse integration levels (Harder et al., 2018). The present study used the IPL-12 scale, which covers each dimension with two items (Harder et al., 2018). Responses to each item are scored between 1 and 5 points, so that a cumulative score on each sub-dimension ranges between 2 and 10 and the overall integration score ranges between 12 and 60. As recommended by Harder et al. (2018), the cumulative scores were then rescaled to provide a standardized IPL Integration Index between 0 and 1. Participants were assigned to two groups of integration whether their overall score was lower or higher than 0.5. Participants (*n* = 4) that did not rate > 10% of the items on the IPL-12 were excluded from the analyses. Further, if participants’ missing items accounted for ≤10% of the IPL-12, the respective items were set to one (lowest value). In the present study, Cronbach’s alpha for the instrument resulted in α_t0_ = 0.82 (*n* = 44) and α_t1_ = 0.77 (*n* = 46).

### 2.4 Data analysis

Version 28 of the Statistical Package for Social Sciences (SPSS; IBM Deutschland GmbH, Ehningen, Germany) was used for all data preparation and statistical analyses. All sum scores and parameters were generated according to the guidelines of the questionnaires and as specified above. All analyses were tested two-sided and with a level of significance of 0.05. As there was no homogeneity of the error variances of emotional distress, longitudinal differences for emotional distress were evaluated by sign tests and *t*-tests. Although RHS difference scores of the total sample (*N* = 46) and cumulative (sub-dimension) integration scores were not normally distributed, t-tests were used, as they are robust with sample sizes >30 (Salkind, 2010), and after non-parametric tests provided the same results. The reported *p-*values for the changes in integration sub-dimensions were Bonferroni corrected. Integration status change over time was examined with a Mixed ANOVA, as homogeneity of the error variances of integration was given. As the Mixed ANOVA was shown to be robust to moderate departures from normality (Salkind, 2010), the Mixed ANOVA for integration was conducted even though one of the four factor groups (integration_t0_: high vs. low and time: assessment_t0_ vs. assessment_t1_) was not normally distributed. The Reliable Change Index (RCI) was calculated to investigate the amount of change of emotional distress and integration in each refugee (Blampied, 2022). Kendall’s tau correlations and multiple hierarchical regression analyses served to delineate the contribution of various post-migration stressors on emotional distress and integration. The categorial variable ‘residence status’ was dummy coded to be included in the regressions and the reference category was secure. The first multiple hierarchical regression analysis targeted emotional distress_t1_ and included in the first block emotional distress_t0_ as the control variable and in the second block integration_t0_, severe physical abuse in childhood, psychotherapy, and residence status_t1_. The second multiple hierarchical regression analysis predicted integration_t1_ with integration_t0_ and length of stay in Germany first entered as control variables. Emotional distress_t0_ and residence statuts_t1_ were entered in a second block. The model fit of the regressions was examined by assessing in which model the Akaike Information Criterion (AIC) was lowest (Akaike, 1987).

## 3 Results

### 3.1 Participants’ current living situation

The time interval between the assessment_t0_ und the assessment_t1_ varied around *M* = 9.3 (*SD* = 0.7) months. *Length of stay_t1_* was *M* = 38.0 (*SD* = 22.3, *RoV* = 10–82) months and *M* = 19.6 (*SD* = 6.0, *RoV* = 14–36) *pandemic months_t1_* (months since the beginning of the COVID-19 pandemic in March of 2020) had passed. Of the total sample (*N* = 46; mean *age_t1_* = 20.8; *SD* = 2.2 years), *n* = 10 had entered Germany as *accompanied minors* and *n* = 14 had entered Germany as *unaccompanied minors*. At the assessment_t0_ 61.0% of participants *did not have parents or siblings living in Germany*. *Years of education_t0_* ranged from 0 to 15 years and participants had visited on average 8.5 (*SD* = 3.4) years of education. Most of the participants were from Afghanistan (32.6%), Syria (17.4%), and Gambia (15.2%). Since their arrival in Germany six participants of 46 in total, and since the assessment_t0_ three of 46 in total reported having been involved in *physical fights*. Seventeen reported having experienced *severe physical abuse in childhood*.

Nine participants had started *therapy* in the *Fearless* project after the assessment_t0_. At the assessment_t1_, six of these were still in therapy, two had ended therapy prematurely after more than six sessions, and one had ended therapy before the sixth session. To control for possible effects of psychotherapy in the following examinations, we categorized these participants in two groups. Research in Germany proposes to classify psychotherapy as “started” when patients have completed at least six sessions (Hiller et al., 2011; Cinkaya et al., 2011). This classification is based on the German health care system where patients first complete up to six probationary sessions before starting a psychotherapy. In these probationary sessions therapists further clarify diagnoses and plan the therapy (Hiller et al., 2011). In accordance, we classified those having completed more than six sessions (*n* = 8) as “having started psychotherapy.” Those that had completed at least six sessions had completed *M* = 33.4 (*SD* = 13.0, *RoV =* 17–54, *n* = 7) therapy sessions at assessment_t1_. Table 1 provides an overview of further details regarding the sociodemographic information of the sample.

**Table 1:** Sociodemographic information of participants at the assessment_t0_ and at assessment_t1_.

Variable	Assessment_t0_	Assessment_t1_
Health insurance card	17.2% (*n* = 29)	63.6% (*n* = 44)
Occupation		
School education/vocational training	55.6%	50.0%
No occupation	20.0%	4.3%
Language courses	15.6%	23.9%
Full- or part-time employment	8.9%	21.7%
Residence status		
Secure (residence permit)	19.6%	41.3%
Partly insecure (first asylum process in progress)	39.1%	26.1%
Not secure (rejected after first or second asylum process)	41.3%	32.6%

If values do not depict the total sample (*N* = 46) the deviating number of n is indicated.

### 3.2 Longitudinal course of emotional distress

Overall, emotional distress decreased over time. Paired t-tests of RHS-scores of the total sample [*t*(45) = −2.45, *p* = 0.018, *d* = 0.36] and of the *n =* 23 participants with RHS_t0_ ≥ 11 [*t*(22) = −4.04, *p* < 0.001, *d* = 0.84] revealed significantly lower RHS-scores at the assessment_t1_ than at the assessment_t0_. This decrease was primarily seen in participants with high distress at assessment_t0_, as the sign test did not confirm significantly different RHS-scores at assessment_t1_ for participants with RHS_t0_ ≤ 11: *p* = 0.115, *n* = 23. The RCI (rounded to +10 or − 10), indicated a significant decrease with a RHS score difference of t1-t0 ≤ −10 and a significant increase with a difference of t1-t0 ≥ 10. Of the 12 participants (25.5%) showing a reliable change from assessment_t0_ to assessment_t1_, the RHS score increased in one and decreased in 11. Table 2 lists mean RHS-scores at assessment_t0_ and at the assessment_t1_ for the total sample (*N* = 46) and subgroups with high vs. low emotional distress at assessment_t0_.

**Table 2:** RHS-Scores at assessment_t0_ and assessment_t1_.

Scale	*n*	Assessment_t0_, *M* (SD; RoV)	Assessment_t1_, *M* (SD; RoV)
Total sample	46	14.67 (12.73; 0–45)	10.91 (9.85; 0–36)
RHS-13_t0_ ≥ 11	23	25.04 (9.84; 11–45)	15.78 (doi:10.37; 1–36)
RHS-13_t0_ ≤ 11	23	4.30 (3.13; 0–10)	6.04 (6.43; 0–32)

If values do not depict the total sample (*N* = 46) the deviating number of *n* is indicated.

### 3.3 Longitudinal course of integration

Overall, the Integration Index increased over time in both groups: While the Mixed ANOVA did not confirm significant interaction of time (assessment_t0_ vs. assessment_t1_) and group (high vs. low integration_t0_), assumed sphericity *F*(1,44) = 2.669, *p* = 0.109, partial η^2^ = 0.057, simple main effects were significant for time [assumed sphericity *F*(1,44) = 31.72, *p* < 0.001, partial η^2^ = 0.419] and group [*F*(1,44) = 86.16, *p* < 0.001, partial η^2^ = 0.662]. The RCI (rounded 0.30 or −0.30) indicated a significant increase in the Integration Index with a difference of t1-t0 ≤ 0.30 in two refugees (4.3%).

Changes per sub-dimension were probed by t-tests and reported *p* values for the sub-dimensions are Bonferroni corrected. Scores for economic [*t*(45) = 2.93 *p* = 0.030, *d* = 0.44], social [*t*(45) = 4.13, *p* = 0.006, *d* = 0.60] and linguistic integration [*t*(45) = 4.62, *p* = 0.006, *d* = 0.68] were significantly higher at the assessment_t1_. Scores for the sub-dimensions psychological [*t*(45) = 1.73, *p* = 0.55, *d* = 0.26], political [*t*(45) = 1.48, *p* = 0.88, *d* = 0.22] and navigational integration [*t*(44) = 1.14, *p* > 0.999, *d* = 0.17] were not significantly different at the assessment_t1_. Table 3 shows an overview of the mean Integration Index at assessment_t0_ and at assessment_t1_, divided by low vs. high integration at assessment_t0_and of the mean Integration Indexes of the sub-dimensions at assessment_t0_ and assessment_t1_.

**Table 3:** Integration Index and sub-dimensions at assessment_t0_ and at assessment_t1_.

Scale	*n*	Assessment_t0_, *M* (SD; RoV)	*n*	Assessment_t1_, *M* (SD; RoV)
Total sample	46	0.40 (0.19; 0.04–0.90)	46	0.49 (0.17; 0.17–0.92)
IIndex_t0_ ≤ 0.5	31	0.29 (0.11; 0.04–0.48)	31	0.41 (0.11; 0.17–0.56)
IIndex_t0_ ≥ 0.5	15	0.61 (0.11; 0.50–0.90)	15	0.67 (0.12; 0.48–0.92)
Economic integration	45	0.35 (0.21; 0.00–1)	46	0.46 (0.22; 0.00–1)
Social integration	46	0.31 (0.31; 0.00–1)	46	0.47 (0.31; 0.00–1)
Linguistic integration	46	0.40 (0.32; 0.00–1)	46	0.56 (0.24; 0.13–1)
Psychological integration	46	0.61 (0.27; 0.00–1)	46	0.67 (0.24; 0.00–1)
Political integration	46	0.20 (0.23; 0.00–1)	46	0.25 (0.28; 0.00–1)
Navigational integration	45	0.53 (0.29; 0.00–1)	46	0.57 (0.30; 0.00–1)

If values do not depict the total sample (*N* = 46) the deviating number of n is indicated.

### 3.4 Associations of longitudinal outcome of psychological distress and integration

Correlational analyses substantiated the preceding results (see Table 4): Emotional distress_t1_ correlated positively with emotional distress_t0_ (*r* = 0.43, *p* < 0.001) and negatively with integration_t0_ (*r* = −0.27, *p* = 0.011) and integration_t1_ (*r* = −0.30, *p* = 0.004). In addition, correlation coefficients signaled an association of emotional distress_t1_ with living conditions such as residence status_t1_ (*r* = 0.25, *p* = 0.037) and psychotherapy (*r* = 0.38, *p* = 0.003). Integration_t1_ correlated positively with integration_t0_ (*r* = 0.62, *p* < 0.001), length of stay (*r* = 0.31, *p* = 0.003) and negatively with residence status_t1_ (*r* = −0.25, *p* = 0.034).

**Table 4:** Kendall’s tau correlations.

	Variables	1	2	3	4	5	6	7	8	9	10	11	12
1	Emotional distress_to_	–											
2	Emotional distress_t1_	0.425**	–										
3	Integration_t0_	−0.251*	−0.267*	–									
4	Integration_t1_	−0.203	−0.302**	0.624**	–								
5	Severe physical abuse in childhood	0.300*	0.188	0.151	0.093	–							
6	Length of stay_t1_	−0.107	−0.014	0.467**	0.308**	0.163	–						
7	Pandemic months_t1_	0.214*	0.090	−0.294**	−0.127	0.022	−0.306**	–					
8	Residence status_t0_	−0.025	0.080	0.009	−0.066	0.031	−0.062	−0.169	–				
9	Residence status_t1_	0.258*	0.247*	−0.256*	−0.251*	0.068	−0.139	0.005	0.442**	–			
10	Psychotherapy	0.429**	0.376**	−0.033	−0.047	0.362*	0.160	0.156	0.031	0.355*	–		
11	Gender	−0.274*	−0.159	0.034	0.102	−0.236	−0.233	0.207	−0.089	−0.135	−0.142	–	
12	Age_t1_	0.022	0.077	0.152	0.101	0.137	0.352**	−0.294**	0.049	−0.023	0.087	−0.406**	–

*p < 0.05, **p < 0.01. These values do depict the total sample (*N* = 46).

### 3.5 Regression analyses

Over and above the course of emotional distress and integration over time, the present analyses aimed at evaluating longitudinal predictors of emotional distress and integration. A hierarchical regression analysis confirmed that *emotional distress_t1_* was best predicted by psychotherapy (*ß* = 0.35, *p* = 0.020), emotional distress_t0_ (*ß* = 0.34, *p* = 0.031) and integration_t0_ (*ß* = −0.29, *p* = 0.043), neither residence status _t1_ nor severe physical violence during childhood reached significance. As illustrated in Table 5, the latter model [*F*(6,39) = 7.255, *p* < 0.001] showed the lowest AIC, explained most variance (adjusted *R^2^* = 0.46) and the change in *F*(2.770) was significant *p* = 0.031. *Integration_t1_* was best predicted by integration_t0_ *(ß* = 0.89, *p* < 0.001). As illustrated in Table 6, the first model [*F*(2,43) = 52.849, *p* < 0.001] explained most variance (adjusted *R^2^* = 0.70) and the change in *F* (0.865) was not significant *p* = 0.467. However, the second model showed the lowest AIC and apart from integration_t0_, no other predictor was significant.

**Table 5:** Regression models of emotional distress_t1_ on emotional distress_t0_, integration_t0,_ severe physical abuse in childhood, psychotherapy and residence status_t1_.

Model	Predictor	*b*	*SE b*	*β*	*T*	*P*	*R^2^*	*AIC*
1.	(Constant)	4.104	1.805		2.274	0.028	0.345	192.928
	Emotional distress_t0_	0.464	0.093	0.600	4.971	<0.001		
2.	(Constant)	13.610	4.321		3.150	0.003	0.455	188.950
	Emotional distress_t0_	0.261	0.116	0.337	2.239	0.031		
	Integration_t0_	−15.484	7.414	−0.293	−2.088	0.043		
	Severe physical abuse in childhood	−1.205	2.606	−0.060	–0.463	0.646		
	Psychotherapy	8.973	3.687	0.349	2.434	0.020		
	Residence status_partly secure_	−5.513	3.056	−0.249	−1.804	0.079		
	Residence status_not secure_	0.175	2.891	−0.008	−0.061	0.952		

The determination coefficient *R*^2^ depicts the adjusted R^2^ for the respective regression model. AIC, Akaike Information Criterion. These values do depict the total sample *N* = 46.

**Table 6:** Regression models of integration_t1_ on integration_t0,_ emotional distress_t0,_ length of stay_t1_ and residence status_t1_.

Model	Predictor	*b*	SE b	*β*	*T*	*P*	*R^2^*	AIC
1	(Constant)	0.200	0.033		6.104	<0.001	0.697	−216.962
	Integration_t0_	0.797	0.095	0.891	8.400	<0.001		
	Length of stay_t1_	−0.001	0.001	−0.078	–0.739	0.464		
2	(Constant)	0.217	0.055		3.958	<0.001	0.694	−213.853
	Integration_t0_	0.781	0.102	0.872	7.633	<0.001		
	Length of stay_t1_	−0.001	0.001	−0.070	–0.615	0.542		
	Emotional distress_t0_	0.000	0.001	0.015	0.160	0.874		
	Residence status_partly secure_	0.000	0.041	0.000	0.004	0.997		
	Residence status_not secure_	−0.048	0.035	−0.137	−1.363	0.181		

The determination coefficient *R*^2^ depicts the adjusted *R*^2^ for the respective regression model. AIC, Akaike Information Criterion. These values depict the total sample *N* = 46.

## 4 Discussion

Emotional distress and integration were assessed in a sample of refugees over 9 months to identify determinants of the longitudinal course of emotional distress and integration. Emotional distress of initially emotionally distressed refugees decreased over time but remained mostly clinically elevated, while emotional distress of refugees with low emotional distress at assessment_t0_ did not significantly change. The most relevant longitudinal predictors of emotional distress were their emotional distress and integration at assessment_t0_, as well as whether they started psychotherapy. Integration improved over time, yet contrary to our expectation, successful integration over time only depended on integration at assessment_t0_. Further, only the sub-dimensions of social, economic, and linguistic integration changed significantly from assessment_t0_ to assessment_t1._

The amelioration of *emotional distress* over time in initially highly distressed refugees is in line with other research in Germany examining refugees over a course of 3 (Nikendei et al., 2019), and 4–10 months (Richter et al., 2018). However, similar to results of previous research, whilst the emotional distress of severely affected refugees improved, it still remained in a clinical range (Nikendei et al., 2019). Examining the course of emotional distress of the sample separated into high vs. low emotional distress at assessment_t0_ underlines the importance of examining mean changes of total samples in more detail. Separated according to their distress level at assessment_t0_, the emotional distress of refugees ameliorated only in those who were initially highly distressed. The most important predictor for refugees’ follow-up emotional distress was their emotional distress at the assessment_t0_. This result is in accordance with research examining refugees in Vietnam over a course of more than 20 years, which found that especially trauma-related mental disorders at arrival, and symptom improvement during the first 3 years of resettlement, were important for positive long-term mental health prognosis after 23 years (Vaage et al., 2010).

Highlighting *integration* as the best acculturation strategy for emotional well-being (Behrens et al., 2015; Han et al., 2016), refugees’ integration at assessment_t0_ was a significant predictor for their emotional distress at follow-up: refugees with higher integration at assessment_t0_ showed less emotional distress at assessment_t1_. These results demonstrate the intertwinement of refugees’ mental health and their integration. Brea Larios et al. (2023) examined Afghan refugees in Norway with a modified version (without the political integration sub-dimension) of the Integration Index. They showed that only psychological integration (sense of belonging) had a significant effect on their emotional distress. Due to our limited sample size, we could not further explore the potentially varying impact of different integration sub-dimensions on refugees’ mental health.

The only significant predictor for refugees’ integration at the assessment_t1_, over and above their emotional distress_t0_, or length of their stay, was their integration at assessment_t0_. Our results emphasize that length of stay may rather act as a marker for other explanatory variables, such as language comprehension. No participant showed a reliable decrease of integration, whilst integration in total and on all sub-dimensions improved. This result gives hope that the right support could enhance integration as well as aid the progress of integration quicker. An increase of integration could be supported with *integration courses*, as already established in Germany. These courses include a German language course as well as information on the legal system, history and culture of Germany (Bundesregierung, 2019). Participation in these courses is offered to/required of refugees with a good chance of staying in Germany (Bundesregierung, 2019), which applied only to a small proportion of our sample. Apart from selective and often delayed access to these courses, only about 50% of the participating refugees successfully pass the exams at the end of the integration courses (Klinger et al., 2019). The high dropout rates from language and integration courses highlight the interplay of refugees’ emotional distress and their integration (German National Academy of Sciences Leopoldina, 2018; Walther et al., 2021). A direct and early support of emotionally distressed refugees even before starting an integration course is needed to enable their participation in integration activities (German National Academy of Sciences Leopoldina, 2018; Walther et al., 2021). Moreover, the courses have been criticized for showing high regional differences regarding the quality of and access to them (Etzel, 2022). Hence, there is a high demand for reforming them, especially since all refugees should have equal access (Klinger et al., 2019; Etzel, 2022).

To our knowledge, our study is the first to examine the longitudinal course of all sub-dimensions of the *Integration Index*. The sub-dimensions of social, economic, and linguistic integration changed significantly from assessment_t0_ to assessment_t1_. Psychological and navigational integration may not have significantly changed because both sub-dimensions were already more elevated at the assessment_t0_ than the social, economic, and linguistic integration sub-dimensions at the assessment_t1_. The only sub-dimension with low scores at assessment_t0_ and assessment_t1_ was political integration. Political integration may only be achieved later in the integration process, which would not be surprising regarding refugees’ restricted possibilities to participate in German politics, e.g., not being allowed to vote (Bundesministerium des Inneren und für Heimat, 2023). In Germany, refugees can mostly engage in political life through non-formal political participation and interviews with refugees highlighted that on top of structural barriers especially an insecure legal status and negative stereotyping composed barriers to refugees’ political participation (Ragab, 2018). Lastly, we want to emphasize that Governmental policies shape and are shaped by public opinions and attitudes, which influence refugees’ opportunities for inclusion and participation (Hynie, 2018). Hence, successful integration of refugees is a two-way street requiring a social context enhancing inclusion and participation (Etzel, 2022).

While *severe physical abuse in childhood* was a cross-sectional predictor for refugees’ emotional distress (Potter et al., 2022), it was not a longitudinal predictor. Similarly, Van Wyk et al. (2012) examined refugees’ PTSD, anxiety, depression and somatization symptoms over the course of 7 months and showed that traumatic events were associated with all mental health symptoms at baseline. However, at follow-up the number of traumatic events did not predict mental health outcomes anymore. Hence, symptoms themselves (Van Wyk et al., 2012) and post-migration stressors (Stuart and Nowosad, 2020) seem to be more relevant for the maintenance of pathology. Aligning with this result, Behrendt et al. (2022) showed that material and social stressors and not stressful life events further exacerbated anxiety and depression symptoms in the long term. Further, physical abuse in childhood, amongst other traumatic experiences, is most likely represented by a basic trauma burden, significantly contributing to emotional distress. If this basic trauma burden is taken into consideration, e.g., by controlling for baseline symptoms as in our study, their incremental predictive power may be reduced.

*Psychotherapy* was included as a control variable in our regression. Refugees with higher levels of emotional distress were more likely to be in psychotherapy in-between both assessments, as psychotherapy was only offered to refugees with high emotional distress at assessment_t0_. In our study, 41.7% of all emotionally distressed participants accepted a treatment offer after assessment_t0_. Other studies reported that 0% (Nikendei et al., 2019) to 75% (Hollifield et al., 2013) of emotionally distressed refugees accepted treatment offers. Most likely, higher emotional distress at the assessment_t1_ resulted from the initially more emotionally distressed refugees participating in psychotherapy. Carlsson et al. (2005) examined refugees after 9 months while they were still in treatment and found no significant changes of mental health symptoms. They concluded that the time frame of 9 months to measure an amelioration caused by treatment was most likely too short, especially because therapies had not yet regularly ended. In accordance with this conclusion, systematic research examining long-term effects of NET found that effects were strongest long-term, e.g., at least 6 months after treatment (Siehl et al., 2020). Of all psychotherapies classified as started, 22.2% ended prematurely, which aligns with a weighted average dropout rate of 19.1% in a meta-analysis on refugees (Semmlinger et al., 2021).

One often researched post-migration stressor is refugees’ *residence status*. While in this research some refugees gained asylum acceptance over the course of 9 months (19.6–41.3%), refugees’ residence status was not a significant predictor for their emotional distress or integration. Schick (Schick et al., 2016) also found that residence status was not predictive of refugees’ emotional distress or integration difficulties over a course of 10 years. Refugees’ residence status may rather act as a marker for other post-migration stressors, such as a different access to health care services, language problems or living arrangements (Bauhoff and Göpffarth, 2018; Toar et al., 2009; Gleeson et al., 2020). Furthermore, whilst residence status might be associated with fears of deportation in the first years after arriving in Germany, over time other factors might become more important for long-term psychological well-being. On average, this sample included refugees who arrived in Germany 2 years prior to our assessment_t0._ At assessment_t1,_ 9 months later, the risk of sudden deportation was even lower. Some of those, whose stay in Germany was refused, were already deported. Hence, in our study residence status might have lost its’ predictive value.

Systematic reviews examining the effects of the *COVID-19 pandemic* during 2020 on refugees showed a negative effect on refugees’ (Sevim et al., 2023) and refugee youths’ mental health (Nearchou et al., 2020). However, a first meta-analysis of longitudinal research on the psychological impact of COVID-19 first lockdowns concluded the impact to be rather small in magnitude and very heterogeneous (Prati and Mancini, 2021). Moreover, research examining a longer time-frame in Danish residents concluded that the COVID-19 pandemic may only have had small long-term effects on mental health (Thygesen et al., 2023). In our research, the predictor pandemic months had an impact at the assessment_t0_ (Potter et al., 2022), but was not significant in the longitudinal analysis. Possibly, the negative impact of COVID-19 and associated isolation is waning over time, now that social interactions have returned to a pre-COVID level.

Some limitations need to be taken into consideration when interpreting the results of our study. A general limitation is that we relied on self-report questionnaires, which provide subjective and not verifiable answers. Further, our sample showed great heterogeneity regarding the participants originating from 13 different countries, their different living situations in Germany, differing lengths of stay, residence statuses as well as current activities. This diversity may limit general conclusions and the representativity of the sample, even though refugee samples are well-known for their heterogeneity (Stenmark et al., 2013; Hecker et al., 2018). Moreover, we had no control group without an offer of psychotherapy. The RHS was translated into many different languages while taking into consideration cultural aspects (Hollifield et al., 2016), nevertheless language- and culture-validated questionnaires were not available for everyone. While statistics on asylum seekers in Germany generally report a high amount of male refugees in the age group 16 to 25 years, as much as 73.1–75.9% (Bundesamt für Migration und Flüchtlinge, 2022), the even higher percentage of male participants in our sample might further limit the generalizability of the results. Due to our limited sample size and cross-sectional results, we did not control for gender or age in the regression analyses because there were no significant cross-sectional predictors (Potter et al., 2022). Moreover, Harder et al. (2018) did not find significant differences in Integration Index scores based on age or gender. Since there is a two-way causality between integration and emotional distress (especially at assessment_t0_), our longitudinal design is limited in regard to causal conclusions on the relationship between emotional distress and integration. Another specificity of refugee samples is a high dropout rate at the assessment_t1_. Our drop-out rate of 44.9% is another limitation even though the rate was in line with drop-out rates ranging from 40.7–71.1% in refugee samples (Borho et al., 2020; Nikendei et al., 2019; Ryan et al., 2008; Kindermann et al., 2020).

Refugees’ emotional distress and their integration are intertwined and hence need to be addressed promptly after refugees’ entry in the host society. As a first step we recommend implementing a mental health screening for all refugees on arrival. Schmidt et al. (2023) have just recently demonstrated the feasibility of such a mental health screening directly at a reception center by combining it with the mandatory physical examination. Further adjustments in the German health care system would include reinforcing education on the treatment of refugee clients in the curricula of psychotherapy training. In addition, therapists providing therapy to refugee clients should be supported by nationwide structural support systems, including financing professional translators, peer counsellors, a referral system for refugee clients, and ensuring adequate supervision. Moreover, access to integration courses needs to be improved, so that all refugees profit from an early access.

## Data Availability

The raw data supporting the conclusions of this article will be made available by the authors, without undue reservation.

## Footnotes

^1^In 2024, this regulation was increased to the first 36-months.

^2^Being aware that refugees and asylum seekers refer to populations with different characteristics, to simplify the terminology we refer to both as refugees.
