# Supplementary material for: Refugees’ integration and emotional distress over the course of 9 months
Source: Front Psychol. 2024 Oct 17;15:1459934. doi: 10.3389/fpsyg.2024.1459934 (PMC11537153; doi:10.3389/fpsyg.2024.1459934)
Supplement: Supplementary file 1 [file Table_1.pdf]

Supplementary Files

Supplementary Table 1

Dropout analysis

Characteristics	Total sample (N = 89)	Follow-up completers (n = 46)	Dropouts (n = 43)	Statistic	p value
Male sex, No. (%)	77 (86.5)	42 (91.3)	35 (81.4)	**χ^2(1) = 1.87**	.171
Family in Germany, No. (%)	37 (42)<br>n = 88	18 (39.1)	19 (45.2)<br>n = 42	**χ^2(1) = 0.34**<br>n = 88	.562
Health insurance card, No. (%)	19 (33.3)<br>n = 57	5 (17.2)<br>n = 29	14 (50)<br>n = 28	**χ^2(1) = 6.88**<br>n = 57	.009**
Severe physical abuse in childhood, No yes (%)	28 (32.2)<br>n = 87	17 (37)	11 (26.8)<br>n = 41	**χ^2(1) = 1.02**<br>n = 87	.313
Violent conflict in Germany, No yes (%)	17 (19.5)<br>n = 87	6 (13)	11 (26.8)<br>n = 41	**χ^2(1) = 2.62**<br>n = 87	.105
Age$_{t0}$, M (SD, RoV), years	19.3 (2.5, 13 – 27)	19.6 (2.4, 14 - 23)	19.1 (2.7, 13 - 27)	t (84.189) = -.681	.498
Length of stay$_{t0}$, *M* (*SD*, *RoV*), months	28.2 (23.1; 2 - 96)	29.2 (22.5, 2 - 74)	27.1 (24.1; 2 - 96)	t (85.410) = -.430	.668
Pandemic months$_{t0,}$ *M* (*SD*, *RoV*)	11.5 (6.1, 5 - 28)	10.9 (5.9, 5 - 28)	12.2 (6.4, 5 - 25)	t (85.020) = .940	.350
Country of origin, No. (%)				**χ^2(3) = 5.15**	.161

Syria	21 (23.6)	8 (17.4)	13 (30.2)		
Afghanistan	21 (23.6)	15 (32.6)	6 (14)		
Gambia	13 (14.6)	7 (15.2)	6 (14)		
Other	34 (38.2)	16 (34.8)	18 (41.9)		
Residence status, No. (%)	*n* = 87		*n* = 41	**$\chi^2(2)$ = 1.12** *n* = 87	.572
Secure	21 (24.1)	9 (19.6)	12 (29.3)		
Partly secure	32 (36.8)	18 (39.1)	14 (34.1)		
Not secure	34 (39.1)	19 (41.3)	15 (36.6)		
Emotional Distress, *M* (*SD*, *RoV*)	15.94 (12.73, 0 – 45)	14.67 (12.73, 0 - 45)	17.30 (12.73, 0 - 42)	*t* (86.596) = .973	.333
Integration, *M* (*SD*, *RoV*)	.38 (.17, .04 - .90)	.40 (.19, .04 - .90)	.36 (.16, .06 - .77)	*t* (86.050) = -.918	.361

Note. *p<.05, **p<.01. If values do not depict the total sample the deviating number of *n* is indicated. In accordance with the handling of missing information in the assessment$_{t1}$ sample, we excluded *n* = 8 from the baseline dropout sample due to too much missing data on one of the key variables and *n* = 1 due to the research’s focus on refugee adolescents. This resulted in a total sample (*N* = 89) of *n* = 46 completers and *n* = 43 dropouts.
